# *S100A14* rs11548103 G>A polymorphism is associated with a decreased risk of esophageal cancer in a Chinese population

**DOI:** 10.18632/oncotarget.20868

**Published:** 2017-09-14

**Authors:** Yang Zhao, Feng Yao, Weifeng Tang, Haiyong Gu, Heng Zhao

**Affiliations:** ^1^ Department of Thoracic Surgery, Shanghai Chest Hospital, Shanghai Jiaotong University, Shanghai, China; ^2^ Department of Cardiothoracic Surgery, Affiliated People’s Hospital of Jiangsu University, Zhenjiang, Jiangsu Province, China

**Keywords:** S100A14, polymorphisms, esophageal cancer, molecular epidemiology

## Abstract

**Objective:**

In China in 2009, esophageal cancer was the fifth most commonly diagnosed malignancy and the fourth leading cause of malignancy-related death. Accumulating evidence indicates that genetic factors might play an important role in esophageal squamous cell carcinoma (ESCC) carcinogenesis.

**Materials and Methods:**

In total, we recruited 629 ESCC patients and 686 controls. Genetic variations in the S100A14, MLH1, SMAD7 and CCL22/MDC genes were measured using the ligation detection reaction method.

**Results:**

When the S100A14 rs11548103 GG genotype was considered as the reference group, the GA genotype associated with decreased risk of ESCC (GA vs. GG: adjusted OR = 0.73, 95% CI = 0.57–0.93, *p* = 0.009). In the dominant model, GA/AA variants were associated with a significantly decreased risk of ESCC compared with the GG genotype (GA/AA vs. GG: adjusted OR = 0.76, 95% CI = 0.61–0.95, *p* = 0.018). Logistic regression analyses showed that the MLH1 rs1800734 C>T, SMAD7 rs12953717 C>T and CCL22/MDC rs4359426C>A polymorphisms were not associated with the risk of ESCC in any of the models tested.

**Conclusions:**

Our findings indicated that, in a Chinese population, rs11548103 might contribute to a decreased risk of ESCC. Further studies are need to confirm these data with results from a lager cohort and different ethnic origins.

## INTRODUCTION

In China in 2009, esophageal cancer (EC) was the fourth leading cause of cancer death and the fifth most common diagnosed malignancy [1]. Esophageal squamous cell carcinoma (ESCC) accounts for more than 90% of EC cases. Single nucleotide polymorphisms (SNPs), as individual genetic risk factors, might play a vital role in ESCC carcinogenesis in addition to certain environmental risk factors [2].

*S100A14* is located on chromosome 1q21 and is one of the least-characterized members of theS100 family [3]. S100A14 is a low molecular weight calcium-binding protein [3, 4]. Since loss of expression or overexpression of S100A14 has been reported in tumors, its functional role has been proposed to be organ-specific and involved in tumorigenesis [5]. S100A14 is also a target for p53 and could alter p53 transactivity and stability, and by regulating matrix metalloproteinase (MMP)2 transcription, S100A14 affects cell invasiveness in a p53-dependent manner [6].

S100 proteins take part in the process of terminal differentiation of the human epidermis [7] and have been implicated in cancer, as altered expression levels of some S100 proteins have been identified to correlate with tumor differentiation, including in ESCC. It has recently been reported that the S100 family member S100A14 plays a role in driving esophageal carcinogenesis, showing that extracellular S100A14 may affect EC cell proliferation and/or apoptosis via interaction with the receptor for advanced glycation end-products (RAGE) [8]. S100A14 can also regulate oral squamous cell carcinoma cell by modulating the expression of MMP1 and MMP9 [9]. One genetic variant of *S100A14* (461G>A, rs11548103) is located in the 5′-untranslated region (UTR) and has been shown to disrupt a p53-binding site. This variant is correlated with decreased expression of *S100A14* both *in vitro* and *in vivo* in target tissues. Additionally, a previous study reported that rs11548103-A was associated with risk for ESCC [10].

*MutL homolog 1* (*MLH1*) is a member of the DNA mismatch repair (MMR) genes, which encode several highly conserved proteins. Deficiency in MMR may play important roles in the etiology of cancer. The rs1800734 (-93G/A) polymorphism in *MLH1* is located in the promoter region, which is responsible for the transcriptional activity of this gene.

SMAD7, an inhibitory SMAD, is a negative regulator of the transforming growth factor-beta (TGF-β) signaling pathway, which promotes the anti-inflammatory roles of TGF-β signaling via binding to the TGF-β-activated kinase (TAK)1-binding proteins that inhibit TAK1, TAB2 and TAB3 [11, 12]. The rs12953717-T allele at *SMAD7* has been associated with increased susceptibility to colorectal cancer (CRC) among both Caucasians and Asians [13].

Macrophage-derived chemokine (MDC), also known as C-C motif chemokine 22 (CCL22), is a chemokine secreted mainly by macrophages [14]. Rs4359426, a variant of *CCL22*, has been shown to associate with over-expression of *CCL22* mRNA and susceptibility to atopic dermatitis in a gain-of-function manner [15].

Genetic variants in *S100A14* (rs11548103G>A)*, MLH1* (rs1800734 C>T)*, SMAD7* (rs12953717C>T) and *CCL22/MDC* (rs4359426C>A) may contribute to the etiology of ESCC. In a hospital-based case-control study including 629 ESCC cases and 686 controls, we performed genotyping and tested the association of these four functional SNPs with ESCC in a Chinese population.

## RESULTS

### Characteristics of the study population

Characteristics of the ESCC cases and controls are summarized in Table 1. According to *χ*^2^ tests, the ESCC cases and controls were adequately matched for age and sex. Meanwhile, we found significant differences in smoking and drinking status between the ESCC cases and controls. Table 2 presents information on *S100A14* rs11548103G>A, *MLH1* rs1800734 C>T, *SMAD7* rs12953717 C>T and *CCL22/MDC* rs4359426 C>A. For these four genotyped SNPs, in our controls, the minor allele frequency (MAF) was very similar to the MAF for Chinese in database. Furthermore, in controls, goodness-of-fit *χ*^2^ tests indicated that the observed genotype frequencies were all in Hardy-Weinberg equilibrium (HWE) for these polymorphisms (Table 2).

**Table 1:** Distribution of selected demographic variables and risk factors in ESCC cases and controls

Variable	Cases (*n* = 629)		Controls (*n* = 686)		*p*^a^
	*n*	%	*n*	%	
Age (years) mean ± SD	62.85 (± 8.13)		62.58 (± 7.89)		0.541
Age (years)					0.155
< 63	310	49.28	365	53.21	
≥ 63	319	50.72	321	46.79	
Sex					0.185
Male	444	70.59	461	67.20	
Female	185	29.41	285	32.80	
Tobacco use					< 0.001
Never	355	56.44	499	72.74	
Ever	274	43.56	187	27.26	
Alcohol use					< 0.001
Never	428	68.04	526	76.68	
Ever	201	31.96	160	23.32	

^a^Two-sided *χ*^2^ test and student *t* test; Bold values are statistically significant (*p* < 0.05).

**Table 2:** Primary information for *S100A14* rs11548103 G>A, *MLH1* rs1800734 C>T, *SMAD7* rs12953717 C>T and *CCL22/MDC* rs4359426 C>A polymorphisms

Genotyped SNPs	S100A14 rs11548103 G>A	MLH1rs1800734 C>T	SMAD7rs12953717 C>T	CCL22/MDCrs4359426C>A
Chromosome	Chr1	Chr3	Chr18	Chr16
Function	UTR-5	UTR-5	Intron	missense
Chr Pos (Genome Build 36.3)	151854964	37009950	44707927	55950234
Regulome DB Score^a^	5	4	5	4
TFBS^b^	Y	Y	—	—
Splicing(ESE or ESS)	Y	—	—	Y
miRNA(miRanda)	—	—	—	—
nsSNP	—	—	—	Y
MAF^c^ for Chinese in database	0.333	0.415	0.183	0.136
MAF in our controls (*n* = 686)	0.323	0.416	0.206	0.149
*p* value for HWE^d^ test in our controls	0.741	0.944	0.889	0.520
Genotyping method^e^	LDR	LDR	LDR	LDR
% Genotyping value	95.29%	96.43%	95.13%	98.63%

^a^ http://www.regulomedb.org/.

^b^ TFBS:transcription factor binding site (http://snpinfo.niehs.nih.gov/snpinfo/snpfunc.htm).

^c^ MAF: minor allele frequency, *S100A14* rs11548103 G>A MAF is in CHB+JPT population.

^d^ HWE: Hardy–Weinberg equilibrium.

^e^ LDR: ligation detection reaction.

### Association between rs11548103, rs1800734, rs12953717 and rs4359426 and risk of ESCC

As shown in Table 3, the GG, GA and AA allele frequencies of rs11548103 were 51.2, 37.8 and 10.9%, respectively, in the ESCC group and 45.5, 44.3 and 10.2%, respectively, in the healthy control group. When the GG genotype was adopted as the reference group, the GA genotype significantly decreased the risk of ESCC (GA vs. GG: adjusted OR = 0.73, 95% CI = 0.57–0.93, *p* = 0.009). In the dominant model, we also found that carriers of the GA/AA variants had a decreased risk of ESCC compared with carriers of the GG genotype (GA/AA vs. GG: adjusted OR = 0.76, 95% CI = 0.61–0.95, *p* = 0.018) (Table 3).

**Table 3:** Logistic regression analyses of associations between *S100A14* rs11548103 G>A, *MLH1* rs1800734 C>T, *SMAD7* rs12953717 C>T and *CCL22/MDC* rs4359426 C>A polymorphisms and risk of ESCC

Genotype	Cases (*n* = 629)		Controls (*n* = 686)		Crude OR (95% CI)	*p*	Adjusted OR^a^ (95% CI)	*p*
	*n*	%	*n*	%				
*S100A14* rs11548103 G>A								
GG	309	51.2	296	45.5	1.00		1.00	
GA	228	37.8	288	44.3	**0.76 (0.60–0.96)**	**0.022**	**0.73 (0.57–0.93)**	**0.009**
AA	66	10.9	66	10.2	0.96 (0.66–1.40)	0.823	0.90 (0.61–1.33)	0.597
AA vs.GA vs.GG								0.064
GA + AA	294	48.8	354	54.5	**0.80 (0.64–0.99)**	**0.044**	**0.76 (0.61–0.95)**	**0.018**
GG + GA	537	89.1	584	89.8	1.00		1.00	
AA	66	10.9	66	10.2	1.09 (0.76–1.56)	0.648	1.04 (0.72–1.51)	0.819
G allele	846	70.1	880	67.7	1.00			
A allele	360	29.9	420	32.3	0.89 (0.75–1.06)	0.185		
*MLH1* rs1800734A>G								
AA	207	33.9	224	34.1	1.00		1.00	
AG	291	47.6	320	48.7	0.98 (0.77–1.26)	0.899	0.92 (0.72–1.18)	0.518
GG	113	18.5	113	17.2	1.08 (0.78–1.49)	0.631	1.03 (0.74–1.43)	0.849
GG vs.AG vs.AA								0.827
AG + GG	404	66.1	433	65.9	1.01 (0.80–1.27)	0.936	0.95 (0.75–1.20)	0.668
AA + AG	498	81.5	544	82.8	1.00		1.00	
GG	113	18.5	113	17.2	1.09 (0.82–1.46)	0.547	1.08 (0.81–1.45)	0.589
A allele	705	57.7	768	58.4	1.00			
G allele	517	42.3	546	41.6	1.03 (0.88–1.21)	0.700		
*SMAD7* rs12953717 C>T								
CC	355	59.2	410	63.0	1.00		1.00	
CT	212	35.3	214	32.9	1.14 (0.90–1.45)	0.266	1.19 (0.93–1.51)	0.162
TT	33	5.5	27	4.1	1.41 (0.83–2.39)	0.201	1.37 (0.80–2.34)	0.255
TT vs.CT vs.CC								0.288
CT + TT	245	40.8	241	37.0	1.17 (0.94–1.47)	0.167	1.21 (0.96–1.53)	0.109
CC + CT	567	94.5	624	95.9	1.00		1.00	
TT	33	5.5	27	4.1	1.35 (0.80–2.27)	0.265	1.29 (0.76–2.19)	0.353
C allele	922	76.8	1034	79.4	1.00			
T allele	278	23.2	268	20.6	1.16 (0.96–1.41)	0.118		
*CCL22/MDC*rs4359426C>A								
CC	461	74.8	491	72.1	1.00		1.00	
CA	138	22.4	177	26.0	0.83 (0.64–1.07)	0.155	0.86 (0.66–1.12)	0.254
AA	17	2.8	13	1.9	1.39 (0.67–2.90)	0.376	1.38 (0.65–2.91)	0.398
AA vs.CA vs.CC								0.217
CA + AA	155	25.2	190	27.9	0.87 (0.68–1.11)	0.265	0.90 (0.70–1.15)	0.391
CC + CA	599	97.2	668	98.1	1.00		1.00	
AA	17	2.8	13	1.9	1.46 (0.70–3.03)	0.312	1.43 (0.68–3.01)	0.343
C allele	1060	86.0	1159	85.1	1.00			
A allele	172	14.0	203	14.9	0.93 (0.74–1.15)	0.496		

^a^Adjusted for age, sex, smoking and drinking status; Bold values are statistically significant (*p* < 0.05).

Logistic regression analyses indicated that rs1800734, rs12953717 and rs4359426 were not associated with the risk of ESCC in any of the models (Table 3).

### Stratification analyses on rs11548103

To evaluate the effects of rs11548103 on ESCC risk according to different age groups, sex, tobacco consumption and drinking status, we performed stratification analyses. A significantly decreased risk of ESCC associated with rs11548103 was evident among younger patients (GA vs. GG: adjusted OR = 0.69, 95% CI = 0.49–0.98, *p* = 0.038), male patients (GA/AA vs. GG: adjusted OR = 0.70, 95% CI = 0.53–0.92, *p* = 0.012) and patients who never drink (GA/AA vs. GG: adjusted OR = 0.72, 95% CI = 0.55–0.94, *p* = 0.017) or smoke (GA/AA vs. GG: adjusted OR = 0.72, 95% CI = 0.54–0.96, *p* = 0.025) (Table 4).

**Table 4:** Stratified analyses between *S100A14* rs11548103 G>A polymorphism and ESCC risk by sex, age, smoking status and alcohol consumption

Variable	S100A14 rs11548103 G>A(case/control) a				Adjusted OR b (95%CI); p; phc				
	GG	GA	AA	GA + AA	GG	GA	AA	GA + AA	AA vs. (GA + GG)
Sex									
Male	221/194	159/200	47/42	206/242	1.00	**0.66 (0.49–0.88);** ***p*: 0.005**; *p*_h_:0.311	0.90 (0.56–1.44); *p*: 0.659; *p*_h_:0.869	**0.70 (0.53–0.92);** ***p*: 0.012**;*p*_h_:0.420	1.09 (0.70–1.72); *p*: 0.699; *p*_h_:0.628
Female	88/102	69/88	19/24	88/112	1.00	0.88 (0.57–1.35); *p*: 0.559;*p*_h_: 0.311	0.92 (0.47–1.81); *p*: 0.808;*p*_h_:0.869	0.89 (0.59–1.33); *p*: 0.565;*p*_h_:0.420	0.97 (0.51–1.86); *p*: 0.937;*p*_h_:0.628
Age									
< 63	154/155	110/149	34/40	144/189	1.00	**0.69 (0.49–0.98);** ***p*: 0.038;** *p*_h_:0.873	0.85 (0.50–1.43); *p*: 0.532; *p*_h_:0.488	0.73 (0.53–1.00); *p*: 0.051; *p*_h_:0.739	1.00 (0.60–1.65); *p*: 0.989; *p*_h_:0.494
≥ 63	155/141	118/139	32/26	150/165	1.00	0.76 (0.54–1.06); *p*: 0.104;*p*_h_:0.873	0.97 (0.55–1.74); *p*: 0.929;*p*_h_:0.488	0.79 (0.57–1.09); *p*: 0.152;*p*_h_:0.739	1.11 (0.64–1.94); *p*: 0.708;*p*_h_:0.494
Smoking status									
Never	186/219	119/206	34/46	153/252	1.00	**0.68 (0.50–0.92);** ***p*: 0.012;** *p*_h_:0.431	0.92 (0.56–1.50); *p*: 0.732; *p*_h_:0.728	**0.72 (0.54–0.96);** ***p*: 0.025**; *p*_h_:0.429	1.09 (0.68–1.75); *p*: 0.730; *p*_h_:0.871
Ever	123/77	109/82	32/20	141/102	1.00	0.84 (0.56–1.27); *p*: 0.412;*p*_h_:0.431	0.97 (0.51–1.85); *p*: 0.932;*p*_h_:0.728	0.87 (0.59–1.28); *p*: 0.475;*p*_h_:0.429	1.06 (0.58–1.95); *p*: 0.853;*p*_h_:0.871
Alcoholconsumption									
Never	217/229	145/220	47/48	192/268	1.00	**0.66 (0.50–0.89);** ***p*: 0.006;** *p*_h_:0.364	0.97 (0.61–1.55); *p*: 0.907; *p*_h_:0.492	**0.72 (0.55–0.94);** ***p*: 0.017;** *p*_h_:0.602	1.17 (0.75–1.82); *p*: 0.498; *p*_h_:0.330
Ever	92/67	83/68	19/18	102/86	1.00	0.93 (0.59–1.47); *p*: 0.747;*p*_h_:0.364	0.80 (0.38–1.67); *p*: 0.551;*p*_h_:0.492	0.90 (0.58–1.39); *p*: 0.638;*p*_h_:0.602	0.83 (0.41–1.67); *p*: 0.601;*p*_h_:0.330

^a^ The genotyping was successful in 603 (95.9%) ESCC cases, and 650 (94.8%) controls for *S100A14* rs11548103 G>A.

^b^ Adjusted for age, sex, smoking status and alcohol consumption (besides stratified factors accordingly) in a logistic regression model.

^c^ *p*_h_ for heterogeneity.

## DISCUSSION

In the present hospital-based case-control study of ESCC, we identified that rs11548103 was associated with decreased risk of ESCC.

Accumulating evidence has demonstrated the importance of the S100 family in cell migration, invasion and cancer metastasis [6]. S100A14, a member of the S100 family, is involved in several vital functional and pathological processes [16]. Additionally, it is predicted to be under tight transcriptional and post-translational regulation [16]. A previous phylogenetic investigation of the S100 family indicated that S100A14 is different from the other members of the S100 family (except S100A13) due to alterations in several key amino acid residues, which are responsible for the binding of calcium, suggesting that the activity of the S100A14 protein is independent of calcium [5]. In addition, functional studies have reported that S100A14 induces cell cycle arrest or apoptosis in ESCC [8, 10] and regulates the cell cycle in a p53- or RAGE-dependent manner.

Chen et al. reported that high levels of S100A14 associated significantly with elevated levels of MMP2 in clinical breast cancer samples with wild-type p53, but not in those with mutant p53 [5]. Although the function of S100A14 in breast cancer remains to be elucidated, it has been suggested that S100A14 binds HER2 and modulates its phosphorylation, leading to HER2-stimulated cell proliferation, indicating that S100A14 may be a functional partner of HER2 in HER2-positive breast tumors [5]. Decreased expression of S100A14 with its genetic variant may be associated with an undifferentiated phenotype and poor prognosis in gastric cancer [17]. In a previous study, rs11548103was demonstrated to diminish a p53-binding site and was correlated with decreased expression of *S100A14* both *in vitro* and *in vivo* in target tissues [10]. Furthermore, a case-control analysis showed that the *S100A14* rs11548103-A allele was associated with susceptibility to ESCC among smokers [10]. However, in our research, we found a protective effect of rs11548103. Additionally, rs11548103 appears to be a functional locus according to a SNP functional prediction website (http://snpinfo.niehs.nih.gov/snpinfo/snpfunc.htm). However, the etiology of rs11548103 is not clearly known and requires further investigation.

Using the Power and Sample Size Calculation program (PS, version 3.0, 2009, http://biostat.mc.vanderbilt.edu/twiki/bin/view/Main/PowerSampleSize), with α = 0.05, the power of our analysis was 0.597 to detect an effect with an adjusted OR of 0.76 in 603 ESCC cases and 650 non-cancer controls.

In this case-control study, there were several limitations. First, the ESCC patients and non-cancer controls were enrolled from local hospitals, where inherent bias may have occurred. Second, the polymorphisms we studied do not provide an extensive view of the genetic variability present. In the future, fine-mapping studies are required. Third, because of the limited sample size and absence of a validation cohort, the statistical power of our study was limited. Fourth, we did not obtain detailed cancer metastasis and survival information, which further restricted the analysis of *S100A14* rs11548103 G>A polymorphism in ESCC progression and prognosis. The actual power might have decreased considerably upon stratification because of the very small sample numbers. Finally, *in vitro* or tissue-specific biological characterizations are required to confirm the current preliminary findings.

In conclusion, our study found that rs11548103 may decrease the risk of ESCC. Tissue-specific biological characterization and a replication study with larger populations are required to confirm our findings.

## MATERIALS AND METHODS

### Ethical approval of the study protocol

We complied with the World Medical Association Declaration of Helsinki regarding ethical conduct of research involving human subjects and/or animals. The review board of Jiangsu University (Zhenjiang, China) approved the present case-control study. Written informed consent was provided by all participants.

### ESCC patients and controls

From two affiliated hospitals (the Affiliated People’s Hospital and the Affiliated Hospital of Zhenjiang, China), between October 2008 and December 2010, 629 cases with ESCC were recruited consecutively. All cases of ESCC were diagnosed by pathological analyses. Patients who previously had a history of malignancy or any metastasized cancer treated with radiotherapy or chemotherapy were excluded. For the control group, 686 non-cancer patients were matched to the ESCC cases with regard to age (± 5 years) and sex. The controls were recruited during the same time period from the two affiliated hospitals of Jiangsu University. Most of the non-cancer controls were being treated for trauma.

Using a pre-tested questionnaire, two trained interviewers questioned each participant personally. The demographic data information (e.g., age, sex) and ESCC-related risk factors (such as smoking and alcohol consumption) were obtained.

### Isolation of DNA and genotyping by ligation detection reaction (LDR)

According to the manufacturer’s protocol, genomic DNA was isolated from whole blood [18]. With technical support from the Biotechnology Company (Biowing Biotechnologies Inc., Shanghai, China), the DNA samples were genotyped using the PCR-LDR method [19]. One hundred sixty (12.17%) were randomly selected for quality control checks and the reproducibility was 100%.

### Statistical analyses

Using *χ*^2^ statistical tests, we tested whether there were differences between cases and controls in the distributions of demographic characteristics (age and sex), selected variables (smoking and alcohol consumption), and the rs11548103, rs1800734, rs12953717 and rs4359426 genotypes. Using logistic regression analyses, the relationship of these four SNPs with risk of ESCC was assessed in terms of crude ORs and ORs adjusted for age, sex and smoking and alcohol consumption. HWE was tested by a goodness-of-fit *χ*^2^ test among the control subjects. All statistical analyses were performed with SAS 9.1.3 (SAS Institute, Cary, NC, USA).

## Abbreviations

LD: linkage disequilibrium
OR: odds ratio
CI: confidential interval
SNPs: single nucleotide polymorphisms
ESCC: esophageal squamous cell carcinoma
